# Coastal sea level projections with improved accounting for vertical land motion

**DOI:** 10.1038/srep16085

**Published:** 2015-11-03

**Authors:** Guoqi Han, Zhimin Ma, Nan Chen, Jingsong Yang, Nancy Chen

**Affiliations:** 1 Northwest Atlantic Fisheries Centre, Fisheries and Oceans Canada, St. John’s, Canada; 2 Second Institute of Oceanography, State Oceanic Administration, Hangzhou, China

## Abstract

Regional and coastal mean sea level projections in the Intergovernmental Panel for Climate Change (IPCC) Fifth Assessment Report (AR5) account only for vertical land motion (VLM) associated with glacial isostatic adjustment (GIA), which may significantly under- or over-estimate sea level rise. Here we adjust AR5-like regional projections with the VLM from Global Positioning Satellite (GPS) measurements and/or from a combination of altimetry and tide-gauge data, which include both GIA and non-GIA VLM. Our results at selected tide-gauge locations on the North American and East Asian coasts show drastically different projections with and without non-GIA VLM being accounted for. The present study points to the importance of correcting IPCC AR5 coastal projections for the non-GIA VLM in making adaptation decisions.

Mean sea level rise is one of the key factors that impact coastal communities under an increasingly warming climate. The rise of mean sea level is mainly responsible for more frequent flooding in many parts of the world in recent decades^1^,^2^. Under the medium-level A1B emission scenario^3^, the IPCC Fourth Assessment Report (AR4) projected global sea level rise of 0.21–0.48 m in the 21^st^ century and of 0.20–0.61 m when considering the dynamic effect of accelerated ice-sheet mass loss^4^. The IPCC AR5 projected global mean sea level rise of 0.36–0.71 m by 2100 under Representative Concentration Pathway (RCP) 4.5 (medium-level emission) and of 0.52–0.98 m under RCP8.5 (high-level emission)^1^. Slangen *et al.*’s projections over the period from 1986–2005 to 2081–2100 are similar to those of IPCC AR5, at 0.54 ± 0.19 m and 0.71 ± 0.28 m under RCP4.5 and RCP8.5 respectively^5^.

Global sea level rise is surely one of the most important climate change indicators. Nevertheless it is the regional and local sea level rise that really matters for adaptation to the sea level rise. Recently, increasing efforts have been made towards understanding past sea level trends and projecting future sea level changes on the regional scale. Local sea level trend can differ significantly from that of the global mean^6^. An important distinction to make between the regional and global mean sea level is that the former is usually defined as the mean sea level relative to land (hereafter MRSL) and thus directly influenced by the local land motion in the vertical. The projected MRSL changes by Slangen *et al.*^7^ vary spatially from −3.91 to 0.79 m under A1B in the 21^st^ century. IPCC AR5 projections under RCP4.5 vary spatially from −1.74 to 0.71 m by 2100 relative to 1986–2005. Slangen *et al.* found regional variations in sea level change up to 30% above and 50% below the global mean^5^. Han *et al.* showed drastic differences in sea level trends along Atlantic Canada in the past 50–100 years^8^. Their sea level projections for Atlantic Canada in the 21^st^ century vary significantly with location, from much above the global mean sea level rise to no rise at all, mainly attributable to the VLM associated with GIA.

The IPCC AR5 pointed out the importance of accounting for the non-GIA VLM but did not include it in projections provided^1^. In the present study we show that failing to account for the non-GIA VLM may significantly underestimate or overestimate future coastal sea level rise, at selected tide-gauge locations on the North American and East Asian coasts (Fig. 1). We argue that while the IPCC AR5 regional and coastal projections provide general guidelines on future sea level rise, coastal communities should adjust these projections by accounting for the non-GIA VLM in making adaptation decisions.

The regional secular sea level change is influenced by oceanographic adjustment, glacier and ice-sheet melt in response to the present climate change, as well as by GIA to the last glacial maximum^7^ and to the Little Ice Age in some areas. The net GIA effect is the combination of both VLM and a change of the sea surface topography itself. Changes in terrestrial water storage such as ground water extraction and reservoir construction and in sediment and tectonic movements may contribute significantly to MRSL change locally^1^. VLM can be obtained using the Global Positioning Satellites (GPS) measurements^9^,^10^. It can also be estimated from a combination of tide-gauge data and satellite altimetry measurements^8^. GPS-corrected geocentric sea level trends over the past decades show reduced dispersion both on the global and regional scales compared with those corrected with GIA model output^9^,^10^. In the present study, we correct Slangen *et al.*’s^5^ AR5-like regional sea level projections at selected tide-gauge stations, using VLM from GPS and/or from a combination of altimetry and tide-gauge data, which includes both GIA and non-GIA effects. The exact projection period is 95 years from 1986–2005 to 2081–2100. To facilitate comparison, both VLM and sea level change over a period of 95 years are presented hereinafter.

## Results

At Sept-iles and Nain, the GIA model indicates land uplifts of 0.03 and 0.10 m^11^. The geocentric sea level changes are 0.25 ± 0.07 (±standard error) and 0.34 ± 0.05 m based on satellite altimetry. The VLMs derived from a combination of satellite altimetry and tide-gauge (ATG) data are 0.46 ± 0.11 and 0.46 ± 0.48 m at the two sites, consistent with GPS measurements of 0.47 ± 0.06 and 0.44 ± 0.06 m^12^,^13^.

At Neah Bay, the GIA model produces a subsidence of −0.11 m^11^; while the ATG estimate indicates uplift of 0.21 ± 0.16 m, substantially closer to the GPS value of 0.36 ± 0.03 m^10^. The land uplift at Neah Bay is mainly associated with tectonic movement^14^. At New York, the GIA model shows land subsidence of −0.18 m, which agrees reasonably well with the ATG estimate of −0.21 ± 0.23 m. The geocentric sea level rise is 0.18 ± 0.07 m based on satellite altimetry. At Grand Isle, the GIA model VLM is −0.10 m; while the ATG estimate is −0.49 ± 0.12 m, consistent with an averaged GPS value of −0.49 ± 0.07 m from four nearby locations^15^. The large land subsidence may be attributed to the response to Holocene sediment loading at time scales of 100–1000 years^15^. At Galveston, the GIA model indicates a land subsidence of −0.09 m; while the ATG estimate is −0.25 ± 0.13 m, closer to the GPS value of −0.56 ± 0.03 m. The substantial land subsidence is mainly caused by ground water pumping and also by oil and gas extraction^16^.

At Jeju, Peltier’s^11^ model indicates that the GIA process results in small land uplift of 0.04 m. The geocentric sea level from satellite altimetry increases by 0.14 ± 0.09 m. The VLM derived from the ATG is −0.62 ± 0.11 m, which is a significant deviation from estimates by Peltier’s^11^ GIA model output. The large subsidence is associated with other factors instead of GIA, possibly ground water extraction^17^.

There are significant differences in the 21^st^ century sea level projections with and without non-GIA VLM accounted for under RCP8.5, except for New York (Fig. 2). At Sept-iles and Nain, the ATG-based projections are consistent with the GPS-based projections. However both of them are significantly different from the projections with the GIA-model VLM that overestimate sea level rise by about 0.4 m. At Grand Isle, the projections with the GPS or ATG VLM are about 1.1 m, 0.4 m greater than the projected rise with the GIA-model VLM. At Galveston, the projected rise with the GIA-model VLM is 0.5 m smaller than that with the GPS VLM. At Neah Bay, the ATG-based rise is much closer to the GPS-based than the projected rise without non-GIA VLM accounted for. At Jeju, the projected sea level rise based on the ATG-derived VLM is 1.25 m, nearly twice as large as that based on the GIA-model VLM.

The 21^st^ century sea level projections under RCP4.5 show exactly the same differences between the GPS- or ATG-based calculations and the GIA-model-based ones (Fig. 3). The projected rise is smaller and so is the uncertainty under RCP4.5 than RCP8.5.

## Conclusions

We have projected relative sea level rise at selected tide-gauge stations along the North American and East Asian coasts under RCP8.5 and RCP4.5, by accounting for non-GIA VLM, which was not included in the IPCC AR5^1^ or other well-recognized projections^5^. Our projections are significantly different from the estimates based on the IPCC AR5 approach at these sites where there are large non-GIA VLM, except for New York. In addition to the aspects emphasized in literature such as understanding dynamical interactions between ice sheets and oceans^18^, the present study points to the importance of accounting for non-GIA VLM in projecting sea level rise regionally and locally. The projections with improved accounting for the VLM effect may substantially impact the time of emergence for coastal sea level change^19^. Therefore, while the IPCC AR5 regional and coastal projections are useful for general guidelines, coastal communities should adjust IPCC projections by accounting for the non-GIA VLM in making adaptation decisions. The present study also points to the need for the IPCC Sixth Assessment Report to include the non-GIA VLM for sea level projections at tide-gauge stations, by using VLMs from available GPS measurements and from the combination of tide-gauge and altimetry data.

The present study clearly demonstrates the impacts of accounting for non-GIA VLM in regional/local sea level rise projections. For regions along passive margins, such as the eastern coast of North America, it is appropriate to use GPS data over the relatively short duration as a proxy for VLM over the present projection period. For regions that experience earthquake cycle deformation, such GPS VLM may partly (or mostly) represent a transient deformation and thus may not be suitable for use in long-term projections. Further adjustment may be needed to correct for the transient deformation such as postseismic motion, to mitigate the effect of the transient deformation on long-term projections.

## Method

For the demonstration purpose, we have selected the tide-gauge sites that have relatively large VLM but for different mechanisms. They are Sept-iles and Nain along the Canadian coasts, Neah Bay, New York, Grand Isle, and Galveston along the US coasts, and Jeju along the East Asian coasts.

**Figure 1:**
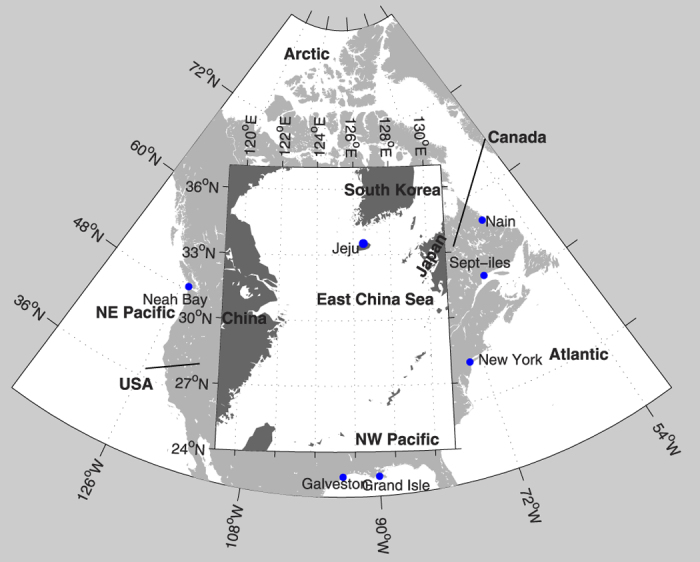
Map of the study regions, with the locations of tide gauges (blue dots). This map is created using MatLab.

**Figure 2:**
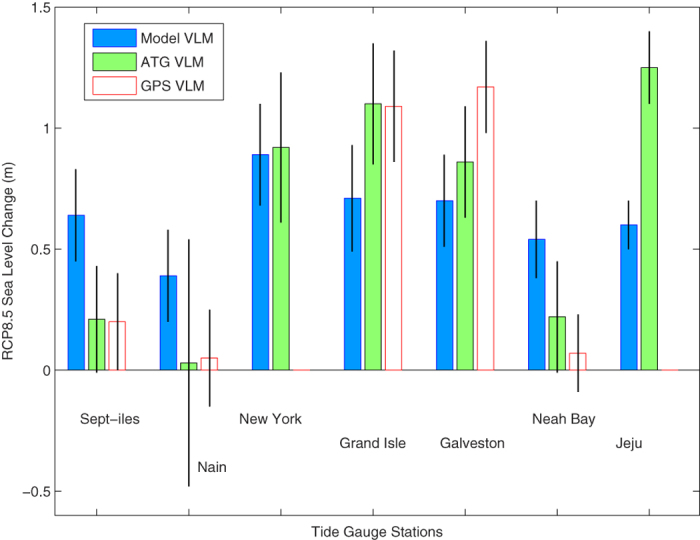
Projected total MRSL changes (vertical bars) ± standard errors (vertical lines) between 1986–2005 and 2081–2100 under RCP8.5, with three different ways of accounting for VLM: Peltier’s GIA model output (Model VLM), ATG-based estimates (ATG VLM) and GPS-based measurements (GPS VLM).

**Figure 3:**
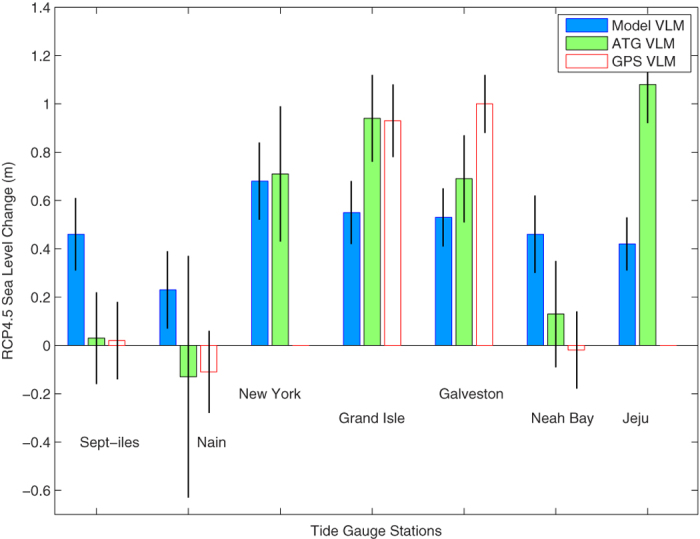
Same as Fig. 2 but under RCP4.5.

We have used annual-mean sea level data at the tide gauge stations (Fig. 1), obtained from the Permanent Service for the Mean Sea Level (PSMSL, http://www.psmsl.org/) over 1993–2012, except for Sept-iles over 1993–2011 and for Nain over 2002–2011. Linear trends of the MRSL and associated standard errors are derived from the tide-gauge data using the least squares fit.

We have used weekly sea surface height anomalies from 1993 to 2002 generated by AVISO (Archiving, Validation and Interpretation of Satellites Oceanographic data) (http://www.aviso.oceanobs.com/en/altimetry/index.html), an objectively mapped product of TOPEX/Poseidon, Jason-1, Jason-2, ERS-1, ERS-2, Geosat-Follow-on and Envisat altimeter data, with a 1/3^o^ Mercator projection grid^20^. The geocentric sea level as measured by satellite altimetry is not influenced by the local land motion. All standard corrections were made by AVISO to account for wet troposphere, dry troposphere, and ionosphere delays, inverted-barometer responses, sea state bias, and ocean, solid earth and pole tides. Linear trends and associated standard errors are derived from the altimetric data using the least squares fit.

We use the GIA model (ICE-5G, VM2) results of Peltier^11^, which include the present-day VLM and the net MRSL change associated with the GIA in a 1^o^ longitude by 1^o^ latitude grid. Following Han *et al.*^8^, we infer the rate of the vertical land motion (VLM) by subtracting the altimetric sea level trend from the trend derived from the tide-gauge data during the same period. The standard error associated with the ATG VLM is estimated as the root-sum-square of the standard errors associated with the altimetric and tide-gauge rates. Note that the ATG estimates include any VLM caused by other factors, in addition to the GIA. We also use the VLM derived from the Global Positioning System (GPS) at Sept-iles, Nain^12^,^13^, Neah Bay^10^, Grand Isle^15^, and Galveston^10^, where the GPS data have a duration of 5–10 years. At Grand Isle, the GPS VLM is estimated by averaging GPS measurements at four nearby locations^15^. The GPS data used in this study are from different sources and thus may not be processed in a consistent manner. Nevertheless, since the present study chooses the sites where the GPS VLM estimate is much larger than the error estimate the processing inconsistency would not affect our conclusions.

We have used Slangen *et al.*’s MRSL projections between 1986–2005 and 2081–2100^5^, under IPCC AR5 emission scenarios RCP8.5 and RCP4.5. Their projections include the steric and dynamic ocean effect from an ensemble mean of 21 CMIP5 climate model output (see their Online Resource Table 1), land-ice melt effect, Peltier’s GIA model output^11^, and effect of ground water depletion. Slangen *et al.*’s^5^ results are linearly interpolated to the tide-gauge locations. The ATG-derived and/or GPS-measured VLMs are also used to replace the GIA VLMs at tide-gauge stations to produce projections that account for non-GIA VLM. The standard errors for total projections are calculated as the root-sum-square of the uncertainties in the ocean and land-ice effects, as well as the errors in the VLM for the ATG- or GPS-based projections.

## Additional Information

**How to cite this article**: Han, G. *et al.* Coastal sea level projections with improved accounting for vertical land motion. *Sci. Rep.* **5**, 16085; doi: 10.1038/srep16085 (2015).
